# Selenium: From Redox Signaling to Interactions with the Gut Microbiome

**DOI:** 10.1007/s12011-026-05132-3

**Published:** 2026-05-01

**Authors:** Marek Kieliszek

**Affiliations:** Department of Food Biotechnology and Microbiology, Institute of Food Sciences, Warsaw University of Life Sciences—SGGW, Nowoursynowska 159 C, Warsaw, 02-776 Poland

**Keywords:** Selenium, Selenoproteins, Redox signaling, Microbiome, Oxidative stress, Gastroenterology

## Abstract

Selenium is an element that plays a crucial role in the proper functioning of the body. It is a component of selenoproteins, which exhibit strong antioxidant properties. This allows it to neutralize reactive oxygen species and protect cells from oxidative stress. It also plays a crucial role in supporting the proper functioning of the immune system. In this context, particular importance is attributed to its influence on the Th1/Th2 immune response and the activity of T lymphocytes and NK cells. There is a mutual relationship between selenium and the intestinal microbiota. Microorganisms in the gastrointestinal tract participate in the accumulation, transformation, and differentiation of selenium’s chemical forms. These processes influence selenium’s bioavailability and its activity in the host organism. The development of metagenomic methods has enabled the identification of specific selenium-dependent metabolic pathways within the microbiome. This represents an important research direction in the development of this field of biotechnology. In turn, appropriate selenium levels and selenoprotein activity influence the composition of the intestinal microbiota and the metabolite profile it produces. It is worth emphasizing that in the context of the development of microbiome engineering, there are also emerging concepts of designing probiotics capable of controlled selenium biotransformation. The beneficial properties of selenium for organisms depend on its appropriate chemical form and dose. It is worth noting that selenium deficiency can impair the antioxidant system, leading to a redox imbalance. Such processes can weaken the integrity of the intestinal barrier, leading to the development of various gastrointestinal diseases. Therefore, the interaction with intestinal microflora is such a crucial element of selenium’s action. Microorganisms inhabiting the digestive tract participate in the processes of accumulation and transformation of various chemical forms of this element. These biochemical properties of microorganisms are crucial for the bioavailability of selenium in the human body. Therefore, the appropriate form of selenium is crucial for the proper functioning of the intestinal barrier. This article discusses the importance of selenium in redox processes and in the function of the gut microbiota. It highlights the potential role of this element in the prevention and treatment of gastrointestinal diseases. Future research should focus on further understanding these interactions and developing targeted approaches that utilize selenium-dependent pathways to restore intestinal homeostasis.

## Introduction

**Fig. 1:**
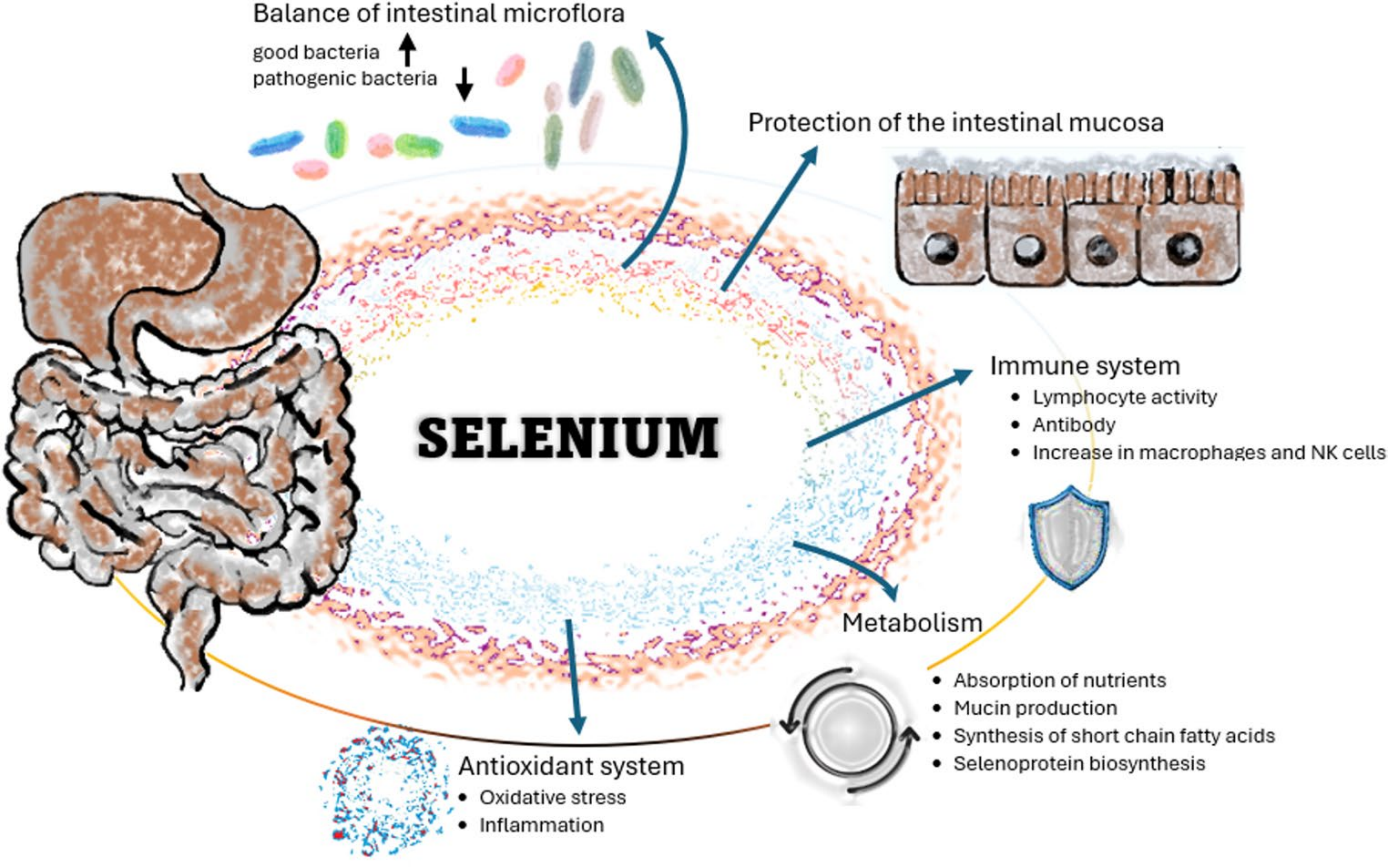
The effect of selenium on the digestive tract and intestinal microflora

Selenium (Se) is a key trace element of great importance for human health [1]. This element participates in regulating intestinal immunity and maintaining gastrointestinal homeostasis. Furthermore, selenium plays a crucial role in modulating interactions among the microbiota, intestinal barrier integrity, and the human immune response [2–4]. It is worth emphasizing that both deficiency and excess of this element can affect the proper functioning of the gastrointestinal tract. Therefore, maintaining its optimal concentration in the body is crucial. This is also crucial for the appropriate functioning of the immune system and maintaining the integrity of the intestinal barrier [5, 6]. Furthermore, selenium is a significant factor determining overall health and a potential element of preventive strategies for various pathological conditions, such as: Crohn’s disease [7], enteritis [2], inflammatory bowel disease [8], cancer diseases [6, 9]. The mechanisms of action of this element primarily include strengthening endogenous antioxidant defense systems and regulating the activity of immune system cells [10, 11] (Fig. 1). However, it is worth emphasizing that the chemical form of selenium used and its concentration are crucial in this regard. According to the European Food Safety Authority (EFSA), the World Health Organization (WHO), and the Institute of Medicine (IOM), selenium can be supplemented in appropriately defined doses [12, 13]. For adults, the recommended daily intake of this element should range from 40 to 70 µg/day [12, 14]. It is worth emphasizing that in specific physiological conditions (e.g., illness, pregnancy), this requirement may be higher [15, 16]. To this end, the maximum amount of the element that can be considered safe for long-term use must be carefully monitored. Furthermore, it is crucial to consider the possibility of disturbances in its absorption processes. The latest data presented by EFSA showed that the Upper Intake Level for adults is 255 µg of selenium per day [17]. Exceeding this value may be associated with the risk of selenosis [18]. This pathological condition manifests itself through nervous system disorders, hair loss, brittle nails, and metabolic dysfunctions [18, 19]. For children and adolescents, the upper limits of selenium intake depend on age and body weight. It is worth emphasizing that supplementation with this element requires caution.

Selenoproteins, including glutathione peroxidases (Gpx) and thioredoxin reductases (TrxR) [20, 21], play a special role in the body’s defense processes. They participate in neutralizing reactive oxygen species [22, 23], thereby reducing oxidative stress and inflammation in the intestines. It is worth emphasizing that these antioxidant enzymes help maintain the balance of intestinal microorganisms and support the proper functioning of the intestinal barrier [5, 6, 24]. Furthermore, selenium deficiency can lead to redox imbalance [25, 26], increased inflammatory response, and impaired immune mechanisms [15]. Such processes increase susceptibility to infections and diseases. Increasing evidence from the literature indicates that selenium status directly affects the composition and function of the intestinal microbiome [27–29]. It is worth emphasizing that in the work by Wang et al. [27], special attention was given to the ability of intestinal microorganisms to biotransform selenium. The authors described that gut microbiota participates in digestive processes and actively transforms various forms of selenium. It is worth noting that numerous microorganisms have been studied to date for their effect on selenium function and metabolism. These include *Bacillus subtilis* [30], *Bifidobacterium longum* [31], *Akkermansia muciniphila* [32], *Ruminococcaceae*, *Lactobacillus* [6] and *Escherichia coli* [33], which serve as important research models for studying interactions between selenium and the gut microbiota [34]. Furthermore, it was emphasized that microbial activity also influences the profile of metabolites produced, including short-chain fatty acids, which play a significant role in the host organism’s functioning. The authors’ proposed approach to assessing selenium bioavailability deserves special attention. It takes into account not only its direct absorption in the gastrointestinal tract but also transformations involving the intestinal microbiota. Thus, the intestinal microbiota is a key factor in determining the action of this element, and its role should be an integral part of interpreting research results on its impact on health. Therefore, this element is essential in strengthening intestinal immunity and immunological defenses in the human body.

## The Role of Selenium In Regulation of Redox Signaling

Selenium, as a component of selenoproteins, participates in numerous physiological functions in the human body [35]. Its unique properties contribute to the regulation of redox reactions [36] in cells and the removal of reactive oxygen species (ROS) [37, 38]. This is of great importance in the human intestinal system. The conversion and transformation of selenates and selenites in eukaryotic cells, involving glutathione systems and various enzymes [39, 40], result in a gradual change in the oxidation state of this element. These processes result in the formation of a key compound, hydrogen selenide (H_2_Se) [41]. It is the basic substrate for the synthesis of all forms of selenium [42]. It is then metabolized into organic forms of selenium. An example of such a compound is selenocysteine (SeCys). This amino acid is formed as a result of the transformation of selenomethionine (SeMet) by the enzyme cystathionine γ-lyase [43, 44]. In subsequent stages of selenium transformation, it is incorporated into proteins as selenocysteine, the 21st amino acid [45]. The redox properties of individual selenoproteins, arising from the presence of the selenocysteine residue in their active sites, help regulate oxidative stress in cells [46, 47]. It is worth noting that the selenium atom is characterized by a lower redox potential and higher nucleophilicity compared to the sulfur present in the amino acid cysteine [48, 49]. Such properties enable a rapid and reversible transition between the reduced form (-SeH) and the oxidized form (–SeOH, –Se–S–, or –Se–Se–) [50–52]. It should be emphasized that the presented mechanism is the basic catalytic property of glutathione peroxidases and thioredoxin reductase. In the intestine, these reactions lead to the reduction of lipid peroxides and hydrogen peroxide via glutathione or thioredoxin oxidation. Moreover, such mechanisms influence the regeneration of reduced cofactors, involving NADPH. Selenium redox metabolism alters the intensity of oxidative stress in intestinal epithelial cells [53, 54]. The ability to change the redox potential is based on the oxidation of selenocysteine residues [23, 55, 56], which play a key role in regulating the activity of proteins involved in maintaining redox homeostasis. This mechanism enables the adaptation of individual cells’ antioxidant systems to changing conditions of oxidative stress. Therefore, maintaining a balance between free radical production and the body’s antioxidant defenses is crucial for the proper functioning of the intestinal system.

At the cellular level, the redox regulation mechanisms of selenium and selenoproteins in the intestinal system [57] are closely integrated with enterocyte metabolism and oxidative stress signaling [26]. The high metabolic activity of enterocytes, resulting from their constant contact with dietary components and the intestinal microbiota, can lead to the formation of significant amounts of reactive oxygen species. Therefore, selenium in the form of hydrogen selenide participates in the biosynthesis of individual selenoproteins [44, 58] which neutralize excess reactive oxygen species. Within the cytoplasm and mitochondria, glutathione peroxidases catalyze the reduction of peroxides [59], protecting cell membranes and mitochondria from lipid peroxidation. This is crucial because it influences the proper functioning and proliferation of intestinal epithelial cells. Individual selenoproteins can regulate the redox state and the activity of transcription factors: nuclear factor κB (NF-κB) and activator protein 1 (AP-1) [57, 60], which are sensitive to oxidative processes. In the case of selenoproteins (selk, sels) located within the endoplasmic reticulum [61] they participate as markers of endoplasmic reticulum stress [62]. Such processes are crucial for maintaining the integrity of the intestinal barrier.

The effect of selenium on the activity of the transcription factors NF-κB and AP-1 is particularly important in regulating signaling pathways [63, 64]. Both of these factors are involved in controlling the inflammatory response, cell proliferation, and cellular stress response. It is worth noting that NF-κB remains inactive in the cytoplasm, bound to the IκB inhibitor [65]. Increased concentrations of reactive oxygen species (ROS) activate IKK, leading to the phosphorylation and degradation of IκB, and consequently, NF-κB translocation to the cell nucleus [66]. By increasing the activity of antioxidant enzymes such as glutathione peroxidases and thioredoxin reductases, selenium limits the excessive accumulation of ROS, thus inhibiting the overactivation of this pathway. Moreover, the thioredoxin system, dependent on thioredoxin reductase (TrxR), directly influences the redox state of cysteine residues in NF-κB subunits, modulating their ability to bind DNA and regulate transcription [67]. Similar relationships are observed in AP-1, which forms a dimeric complex with proteins from the Jun, Fos families and ATF (activating transcription factor) [68, 69]. Its activity is also strongly dependent on the oxidoreductive conditions in the cell [70]. Reversible modifications of cysteine residues in the DNA-binding domains play a key role here. Selenium, by maintaining the appropriate redox potential via the glutathione and thioredoxin systems, indirectly regulates AP-1 activity, thereby influencing the expression of genes associated with proliferation, differentiation, and the inflammatory response [23]. Another important element of this regulatory network is the transcription factor Nrf2, a key regulator of the cellular antioxidant response. Nrf2 was first identified in 1994 [71]. Under conditions of oxidative stress, its activation leads to increased expression of genes encoding antioxidant enzymes. Due to the high efficiency of these enzymes in neutralizing reactive oxygen species (ROS) and in response to exogenous stresses, Nrf2-mediated antioxidant activity plays a key role in cell protection [72]. Under homeostatic conditions, Nrf2 is bound to the Keap1 protein in the cytoplasm and undergoes continuous ubiquitination and proteasol degradation [73, 74]. Increased oxidative stress leads to the modification of redox-sensitive cysteine residues in Keap1, resulting in the release of Nrf2 and its translocation to the cell nucleus [75]. There, it binds to ARE (antioxidant response element) sequences [76, 77], initiating the expression of genes encoding detoxification and antioxidant enzymes. Selenium influences the availability of glutathione and the efficiency of the thioredoxin system, two key systems that help maintain oxidative balance in the cell. This allows it to indirectly regulate the activity of the transcription factor Nrf2 and help the cell better respond to oxidative stress. As an important regulator of redox balance and cell signaling, selenium influences the activity of pathways such as NF-κB, AP-1, and Nrf2 [78]. Consequently, it plays a crucial role in maintaining the balance between oxidative stress, inflammation, and cellular defense mechanisms.

In the human intestinal system, regulation of cellular redox levels via selenoproteins [79] is closely linked to the function of specialized molecular transporters. These biological structures influence the bioavailability of selenium, its intracellular distribution, and participation in specific redox pathways. In the intestinal lumen, inorganic forms of selenium (selenates and selenites) are absorbed through the apical membrane of enterocytes via nonspecific anion transporters. This process occurs, among others, via sulfate cotransporters from the SLC26 family [80], which recognize selenium compounds due to their chemical similarity to sulfate anions [81]. This mechanism highlights the critical role of common transport pathways for selenium and sulfur in regulating the bioavailability of this element in the gastrointestinal tract [82]. It is also worth emphasizing that selenium plays a key role in preventing the development of intestinal diseases [57].

In contrast, selenium in its organic form (e.g., selenomethionine and selenocysteine) can be absorbed via amino acid transporters (the *B⁰AT*1 system encoded by the *SLC6A19* gene) and L-type Amino Acid Transporters (LAT-type transporters). These mechanisms enable efficient selenium transport into the enterocyte cytoplasm. After crossing the cell membrane, this element participates in reactions closely linked to proteins that limit the cytotoxicity of reactive metabolic species. The distribution of selenium between subcellular compartments is indirectly regulated by the glutathione and thioredoxin transport systems [83–85]. Selenium export from enterocytes to the circulatory system occurs primarily through the basolateral membrane [82, 86] It is coupled to the synthesis of selenoprotein P (SELENOP), which serves as the leading selenium transporter in plasma [60, 87]. Selenoprotein P, synthesized in enterocytes and the liver, contains 10 selenocysteine residues [88] and enables the distribution of this element to peripheral tissues via endocytic receptors, including apoer2 and megalin [89]. At the cellular level, molecular transporters interact with redox mechanisms, influencing changes in selenium absorption, storage, and release in response to the cellular oxidative status. Therefore, selenium transport is part of a dynamic regulatory network that integrates redox signaling, cellular metabolism, and intestinal barrier function [2, 90].

## Selenium and Gut Microbiome

The gut microbiota is an ecosystem of individual microorganisms that inhabit the human gastrointestinal tract [91]. It is an integral element of the gut-brain axis [92]. It is worth noting that the gut microbiota is a key regulator of specific metabolic processes, immunity, and intestinal barrier function [93, 94]. Much of the literature indicates [95, 96] that the host’s selenium status influences the composition and activity of the microbiota. Moreover, intestinal microorganisms and their biochemical properties influence the bioavailability and metabolism of selenium [97].

Intestinal bacteria can actively process selenium and convert it into various biologically active compounds. It is worth noting that selenium reduction by microorganisms occurs via two pathways: assimilative reduction and dissimilatory reduction. The first pathway involves the incorporation of selenium into selenoproteins [98]. Dissimilatory reduction, on the other hand, primarily involves the conversion of selenates and selenites to elemental selenium (Se^0^) [99]. This process is highly specific and dependent on the presence of the SECIS sequence in mRNA [100] and a specialized translation apparatus, enabling the incorporation of selenocysteine, among other things, in place of the UGA codon, which typically functions as a stop codon [101, 102]. The resulting selenoproteins perform important redox functions in maintaining the redox balance of bacterial cells [38, 103]. Among the best-known are bacterial selenium-dependent reductases and dehydrogenases, which enhance the microorganisms’ ability to adapt to the changing conditions of the intestinal environment. Literature data presented by Song et al. [104] indicate that *Enterobacter cloacae* Z0206 is capable of reducing selenite (SeO_3_^2−^) to elemental selenium (Se^0^). The reaction is catalyzed by the enzyme fumarate reductase. This process then leads to the production of selenium nanoparticles (SeNPs). Nitrite reductase is involved in selenite reduction in *Rhizobium selenitireducens* strain B1 [105], while sulfite reductase is responsible for this process in *Providencia rettgeri* HF16-A [106]. However, a study by Butler et al. [107] demonstrated that the bacterium *Thauera selenatis* can reduce selenium via selenate reductase. *Lactobacillus casei* ATCC 393 bacteria also convert selenium compounds using nitrite reductase [99]. This enzyme catalyzes the reduction of selenate to selenite by taking electrons from the Q pool via the diheme cytochrome C (Cytc4). Furthermore, enzymes that use sulfite, arsenate, or nitrite as terminal electron acceptors can be used by microorganisms to detoxify selenium [108]. It is also worth emphasizing that the most common route of selenite reduction in bacteria is the so-called Painter reaction (GSH-mediated reduction). In this reaction, selenite reacts with glutathione (GSH), forming GS-Se-SG, which is then reduced by NADPH and GSH reductase to the unstable intermediate GS-Se⁻. This, in turn, undergoes hydrolysis, resulting in the formation of the element selenium (Se⁰) and reduced GSH [99]. It is worth emphasizing that the gut microbiota influences selenium bioavailability by modulating its absorption and distribution. It is worth noting that not all forms of selenium and their properties have yet been discovered.

Selenium can modulate gut microbial balance. Adequate selenium intake promotes eubiosis, while selenium deficiency can lead to intestinal dysbiosis [109]. These effects result in a weakening of the microbial population producing short-chain fatty acids (SCFA) [110]. Bacteria producing SCFAS, such as *Faecalibacterium*,* Roseburia*, or *Eubacterium* [111, 112], are microorganisms susceptible to oxidative stress and prefer conditions with low oxidation-reduction potential [113]. Selenium deficiency in the host can lead to reduced expression of selenoproteins [114], which increases the concentration of reactive oxygen species in the intestinal lumen and within the intestinal epithelium [115]. Increased oxidative stress alters the intestinal environment by reducing the number of anaerobic bacteria producing SCFAs [116]. It is also worth emphasizing that reduced SCFA production has significant consequences for the host. Butyrate is the primary energy source for colonocytes and acts as a signaling molecule (G protein-coupled signaling receptors (gpcrs), activating GPR41 and GPR43, which are protein-coupled receptors [117]. Furthermore, butyrate inhibits histone deacetylase activity, protecting pancreatic cells and reducing liver fibrosis [118, 119]. SCFA deficiency leads to disorders of epithelial cell differentiation, the anti-inflammatory response, and intestinal barrier function [120, 121]. At the immunological level, reduced butyrate availability may impair regulatory T cell differentiation [122]. This, in turn, promotes the development of a pro-inflammatory response, further perpetuating dysbiosis. It is also worth noting that selenium deficiency disrupts redox signaling and increases activation of inflammatory pathways, such as NF-κb [123, 124]. This leads to increased intestinal permeability and the accumulation of microbial metabolites (including lipopolysaccharide), which intensify the inflammatory response in the intestinal mucosa. The consequence of these processes is an increase in the number of facultatively anaerobic and proinflammatory bacteria [125].

These mechanisms are closely linked to selenium’s role in regulating oxidative stress and the inflammatory response in the intestinal mucosa. Selenoproteins influence the integrity of the intestinal barrier, and their reduced expression promotes increased intestinal permeability and bacterial translocation [57], which is essential in the development of inflammatory bowel diseases, metabolic disorders, and autoimmune diseases. Considering the heterogeneity of populations resulting from dietary, geographic, and genetic differences, as well as from distinct microbiota composition, is crucial to understanding the relationship between selenium and the gut microbiota [5, 126–128]. Precisely determining the chemical form of selenium, the dose, and the duration of exposure is also crucial, as these factors are crucial to various biological processes occurring within cells. In recent years, there has been growing interest in the effects of selenium not only on host cells but also on the microorganisms inhabiting the digestive system. Studies by Kasaikin et al. [114] using animal models demonstrated that a selenium-enriched diet can significantly increase gut microbiota diversity and modify its taxonomic composition. It is worth noting that this depends on the chemical form of selenium and its dose. Mice fed a selenium-rich diet demonstrated increased gut microbiome diversity. The changes presented here directly influenced selenoprotein expression and host antioxidant metabolism. The authors observed that in mice fed a selenium-rich diet, the levels and activity of antioxidant enzymes, such as glutathione peroxidase-1 and methionine R-sulfoxide reductase-1, were higher compared to mice not supplemented with selenium. Similar results were obtained in a study by Ankley et al. [129] on finescale dace (*Phoxinus neogaeu*). The study demonstrated that selenium modulated the microbiome’s hormetic response. It is worth emphasizing that intestinal microorganisms can metabolize selenium in distinct biochemical pathways. Furthermore, different forms of selenium compounds influence the structure of the microbiota by altering their metabolic activity. For example, supplementation with selenomethionine or selenium from selenium yeast in dogs and fish increased the proportion of beneficial bacteria, including *Actinobacteria*, *Lachnospiraceae*, and *Ruminococcaceae.* It is worth emphasizing that clinical data in the scientific literature remain limited. Population studies conducted to date in regions with varying selenium intake indicate that dietary selenium intake may be associated with minor changes in the composition and metabolic activity of the gut microbiome [130]. In another study presented by Zahran et al. [28], the authors examined the effect of selenium nanoparticles added to the feed on the gut microbiome of Nile tilapia. The study found that selenium supplementation significantly affected the functioning of the digestive system and microorganisms. SeNPs modulated the gut microbiota, promoting the growth of beneficial bacteria. Furthermore, the authors observed improved intestinal barrier integrity, as evidenced by increased proliferation of mucus-producing goblet cells and by the expression of genes involved in the immune response. According to data presented by Su et al. [131], supplementing weaners’ diets with selenium-enriched yeast may benefit gastrointestinal tract health during the fattening period. This effect is associated with a reduction in the production of isovaleric acid, a marker of unfavorable fermentation processes, and an increase in the number of *Lactobacillus* bacteria, considered an important element of a healthy gut microbiota. Data presented by Jiang et al. [132] indicate that gut microbiota may influence selenium absorption and utilization. Furthermore, the relationship between selenium and the microbiome works in both directions: selenium influences the composition of the microbiota, and the microbiota may shape its availability to the body. The authors’ research results highlight the significant role of selenium in modulating the microbiome.

In summary, selenium and the gut microbiota are closely linked and jointly influence the proper functioning of the body. Current research shows that both selenium deficiency and excess can disrupt microbiome balance, which, in turn, can promote the development of digestive and metabolic diseases. Therefore, further research is crucial to understand better the composition of the gut microbiota, selenium levels in the body, and the role of selenoproteins in gastrointestinal health and disease. Cohort studies with individual monitoring of selenium metabolism are also particularly important. Equally important are in vitro and in vivo studies that will help us better understand how selenium affects the gut microbiome and its function. A better understanding of these relationships will not only expand our knowledge but also help us develop more effective nutritional strategies and therapies.

## The Importance of Selenium In Gastroenterological Diseases

The gastrointestinal tract is particularly sensitive to oxidative stress. This is due to the intense metabolic processes occurring within its cells. Furthermore, it is worth noting that gastrointestinal cells are constantly in contact with a variety of nutrients, the intestinal microbiota, and antigens. Such situations can further impact the proper functioning of defense mechanisms. Therefore, selenium’s role in antioxidant and immunomodulatory activity is crucial. The biological importance of selenium stems primarily from its presence in selenoproteins [133], which protect gastrointestinal epithelial cells from damage caused by reactive oxygen species. In the context of gastrointestinal diseases, adequate selenium levels are associated with modulation of inflammatory processes and maintenance of intestinal barrier integrity. It is worth emphasizing that a growing body of literature [2, 9] indicates that a deficiency of this element may influence the progression of various diseases, including inflammatory bowel disease, colon cancer, and stomach cancer. Moreover, insufficient selenium levels may lead to reduced selenoprotein activity, potentially participating in the mechanisms of fatty liver pathogenesis [134]. Therefore, understanding the role of selenium in the digestive tract provides a basis for considering its potential use in the prevention and treatment of gastrointestinal diseases.

In inflammatory bowel diseases (Crohn’s disease and ulcerative colitis), reduced serum and tissue selenium concentrations have been observed [135, 136]. It should be noted that the presented information directly correlates with disease activity and the degree of inflammation. It is worth emphasizing that selenium deficiency leads to reduced expression of selenoproteins in the intestinal epithelium [137, 138] and impaired redox-dependent signaling. This promotes intestinal barrier dysfunction and increased intestinal permeability. What is also very important, these processes increase intestinal permeability, enabling the translocation of microbial antigens and the activation of molecular pattern recognition receptors (TLRs and NODs) [139, 140]. The consequence of these processes is the intensification of NF-κb and MAPK signaling pathway activation and increased expression of proinflammatory cytokines (TNF-α, IL-1β, and IL-6) [123, 141, 142]. Therefore, maintaining adequate selenium status promotes the balance between regulatory T cells and Th1 and Th17 [143]. This is crucial for controlling intestinal inflammation.

The importance of selenium is also emphasized in the context of liver diseases, including nonalcoholic fatty liver disease, alcoholic liver disease, and cirrhosis. In these diseases, oxidative stress and chronic inflammation play a significant role in the progression of hepatocyte damage [144, 145]. Reduced selenoprotein activity promotes increased lipid peroxidation and fibrosis [146, 147]. Low selenium levels may contribute to the severity of various liver diseases [148]. Therefore, this element can be considered a potential marker of nutritional status and disease severity [148–150]. Selenium also plays a role in the pathogenesis of gastrointestinal cancers, especially colorectal and gastric cancer [9]. Its protective effect is associated with reduced oxidative stress, modulation of DNA repair mechanisms, and regulation of apoptosis [151–153]. At the same time, it should be emphasized that this effect is strongly dependent on the dose and the patient’s initial selenium status, as both selenium deficiency and excess can lead to adverse disease outcomes [15]. When assessing the importance of selenium in gastrointestinal diseases, it is crucial to consider its chemical form, bioavailability, and complex interactions with other dietary components and the intestinal microbiota [5, 27, 34]. Furthermore, it is crucial to consider the characteristics of the patient population, geographic differences in selenium supplementation, and inflammation. All these aspects are essential to the processes of accumulation and metabolism of this element in the human body.

## Conclusions

Selenium is a key integrator of redox homeostasis, immune regulation, and microbial activity in the gastrointestinal tract. A growing body of research is exploring the role of selenoproteins in cellular antioxidant defense. However, it is essential to remember that the biochemical activity of the gut microbiota, the appropriate dose, chemical form, and availability of selenium influence the metabolic efficiency of the gastrointestinal system. Disturbed selenium status in the human body can increase oxidative stress. This results in epithelial dysfunction and a disruption in the quantitative and qualitative composition of the intestinal bacterial ecosystem. All these aspects contribute to the development of various gastrointestinal pathologies. Future research should focus on selenium speciation analysis, host genomics, and microbiome profiling. Obtaining such research results could enable a precise definition of appropriate selenium supplementation strategies depending on the individual gastrointestinal pathophysiology of a given host. Understanding these relationships is crucial for the potential use of selenium in various medical strategies supporting the treatment of digestive system diseases.

## Data Availability

No datasets were generated or analysed during the current study.
